# Molecular Characterization of Barrier Properties in Follicle-Associated Epithelium of Porcine Peyer's Patches Reveals Major Sealing Function of Claudin-4

**DOI:** 10.3389/fphys.2017.00579

**Published:** 2017-08-14

**Authors:** Judith Radloff, Evgeny L. Falchuk, Alexander G. Markov, Salah Amasheh

**Affiliations:** ^1^Institute of Veterinary Physiology, Freie Universität Berlin Berlin, Germany; ^2^Department of General Physiology, Saint Petersburg State University St. Petersburg, Russia

**Keywords:** claudins, tissue barrier, tight junction, pig intestine, gut-associated lymphoid tissue

## Abstract

The pig represents a preferred model for the analysis of intestinal immunology. However, the barrier of the follicle-associated epithelium (FAE) covering porcine Peyer's patches (PP) has not yet been characterized in detail. This study aimed to perform this characterization in order to pave the way toward an understanding of the functional contribution of epithelial barrier properties in gut immunology. Porcine tissue specimens were taken from the distal small intestine in order to obtain electrophysiological data of PP FAE and neighboring villous epithelium (VE), employing the Ussing chamber technique. Transepithelial resistance (TER) and paracellular fluorescein flux were measured, and tissues were morphometrically compared. In selfsame tissues, expression and localization of major tight junction (TJ) proteins (claudin-1, -2, -3, -4, -5, and -8) were analyzed. PP FAE specimens showed a higher TER and a lower apparent permeability for sodium fluorescein than VE. Immunoblotting revealed an expression of all claudins within both epithelia, with markedly stronger expression of the sealing TJ protein claudin-4 in PP FAE compared with the neighboring VE. Immunohistochemistry confirmed the expression and localization of all claudins in both PP FAE and VE, with stronger claudin-4 abundance in PP FAE. The results are in accordance with the physiological function of the FAE, which strongly regulates and limits antigen uptake determining a mandatory transcellular route for antigen presentation, highlighting the importance of this structure for the first steps of the intestinal immune response. Thus, this study provides detailed insights into the specific barrier properties of the porcine FAE covering intestinal PP, at the interface of intestinal immunology and barriology.

## Introduction

The small intestinal mucosa is exposed to a wide variety of exogenous molecules. Forming the first line of immunological defense, Peyer's patches (PP) play an important role in distinguishing between potentially harmful agents and common food ingredients within the ingesta as a major part of the gut-associated lymphoid tissue (GALT; Jung et al., 2010). The intestinal reaction of the immune system, which includes the transport and presentation of antigens, the stimulation of lymphocytes, and the production of antibodies, is essential for gut physiology. Responsible for the uptake and transport of antigens to the underlying tissue is the overlying follicle-associated epithelium (FAE), which forms a boundary separating the lumen contents and immune cells. Therefore, a tight intestinal barrier and a controlled paracellular permeability are prerequisites for a healthy individual.

The transport of antigens through FAE of PP is an important step for the further initiation of immune reactions. FAE differs from neighboring adjacent villous intestinal epithelium (VE) both structurally and by cell composition: within this cell layer, M cells (or microfold cells) are responsible for the translocation and presentation of antigens to immune cells (Onishi et al., 2007). M cells are abundantly distributed in FAE but not in VE and are able to transport antigens directly to the subepithelial lymphoid follicles (Sakhon et al., 2015) or to proceed and present antigens by using major histocompatibility complex (MHC) II (Allan et al., 1993). Moreover, FAE lacks the subepithelial myofibroblast sheath, the basal lamina of FAE is more porous compared to VE, and the basolateral surface of M cells is enhanced by invagination, promoting a faster translocation of antigens (Neutra et al., 2001; Takeuchi and Gonda, 2004). The presentation of antigens induces the activation of B- and T-lymphocytes in the follicular area of PP (Gebert et al., 1996). Local differences in morphology such as the invagination of the basolateral membrane of M cells, the lack of the subepithelial myofibroblast sheath, and a more porous basal lamina promote a faster response to antigens (Takeuchi and Gonda, 2004). However, the selectivity of transcellular mechanisms, including antigen uptake by dendritic cells or ligand-specific transcytosis mediated via Toll-like receptors, might benefit from a stronger paracellular seal.

Whereas, transcellular translocation has been described in detail, limited information is available regarding the characteristics of the Para cellular pathway and its contribution to the immunological function of PP. A comparison of the trans epithelial resistance (TER) of rabbit PP and VE has revealed a general difference, with markedly higher values in FAE compared with neighboring VE (Kucharzik et al., 2000). Although these general structural properties of PP have been described, information on the Para cellular pathway of the PP epithelium remained limited until our previous study showing the differential distribution of the barrier function in correlation with the expression and localization of tight junction (TJ) proteins in rat PP FAE (Markov et al., 2016). In this context, the functional contribution of single TJ proteins to epithelial barrier physiology has been reviewed in detail recently, highlighting the special role of claudins as a main TJ component (Markov et al., 2015, 2017).

The pig is similar to the human concerning its genomics, anatomy, and physiology (Hart et al., 2007; Swindle et al., 2012). As one of the most commonly used animal in biomedical research, the pig presents an important *in vivo* model for investigating physiological and pathological mechanisms in the cardiovascular, urinary, integumentary, and digestive systems (Swindle et al., 2012). Moreover, the porcine intestine has gained major attention as an important model in infectious diseases (Meurens et al., 2012). This extensive use stands in contrast to the limited information available concerning the porcine GALT system, as no data regarding functional and molecular PP FAE barrier properties is currently available.

Thus, our study has aimed to characterize the barrier properties within porcine PP FAE in order to build a substantiated foundation for the understanding of the contribution of the PP FAE barrier in health and disease.

## Materials and methods

### Tissue preparation

Tissue specimens of seven untreated pigs at the age of 2 months were taken immediately after slaughter. The PP and VE were differentiated visually and taken from the distal small intestine. The samples were further processed and used for Ussing chamber experiments, flux measurements, immunoblotting, and immunohistochemistry, as described below. Tissues for Ussing chamber measurements were transported and stored in warm (37°C) transport buffer solution containing (in mmol·l^−1^): Na^+^ (145.2), Cl^−^ (124.8), K^+^ (5), Ca^2+^ (1.2), Mg^2+^ (1.2), ${\text{HCO}}_{3}^{-}$ (25), H_2_${\text{PO}}_{4}^{2-}$ (0.4), ${\text{HPO}}_{4}^{-}$ (2.4), and D-Glucose (5). The solution was permanently gassed with 95% O_2_ / 5% CO_2_, resulting in a pH of 7.4.

### Morphometry

The mucosal surface of the VE is larger than that of PP because of specific anatomical structures. Whereas the VE is typically organized in crypts and villi, the epithelium covering PP is composed of FAE and intermolecular areas of VE. These differences must be taken into account for immunoblot analysis. The morphology of PP in piglets aged 2 months has previously been researched intensively (Barmann et al., 1997). Subsequently, ratio of FAE to VE in PP was calculated. Since FAE is characterized by a dome-like arch of epithelial cells covering the follicles, cellular content is lower when compared with the meandering VE. This morphological difference was also taken into account by employing a method used previously (Markov et al., 2016). Nuclear DAPI (4′,6-diamidino-2-phenylindole) staining of the PP and VE was analyzed via immunofluorescence microscopy (Leica DMI 6000 B, Leica, Germany). Sample areas were chosen at 20× magnification, whereas the subsequent calculation of the cell count in selected lengths was performed at 63× magnification due to easier visualization.

### Electrophysiology

Tissue was mounted in conventional Ussing chambers and left to calibrate until the electrophysiological values were stable. All electrophysiological measurements were performed under voltage clamp conditions, reporting TER. After 45 min of preincubation with fluorescein, measurements were started, and TER was recorded continuously over a period of 60 min. The experimental buffer contained in mmol·l^−1^: Na^+^ (149.4), Cl^−^ (128.8), K^+^ (5), Ca^2+^ (1.2), Mg^2+^ (1.2), ${\text{HCO}}_{3}^{-}$ (25), H_2_${\text{PO}}_{4}^{2-}$ (0.6), ${\text{HPO}}_{4}^{-}$ (1.2), and D-Glucose (10.0). Buffer was warmed to 37°C and gassed with 95 % O_2_ and 5 % CO_2_ continuously. The vitality of the tissue was tested by using theophylline (10 mmol·l^−1^).

### Measurement of paracellular permeability

Paracellular flux was measured using sodium fluorescein (332 Da) as described previously (Radloff et al., 2017). Fluorescein (100 μmol l^−1^) was added to the apical side of the chamber. After preincubation, basolateral samples were taken every 30 min for 1 h, and the removed volume was replaced with fresh experimental buffer. Samples were measured photometrically at a wavelength of 514 nm by utilizing a plate reader (EnSpire Multimode Plate Reader, Perkin Elmer, USA). The resulting flux and permeability were calculated as reported recently (Radloff et al., 2017). Initially, the measured concentration of sodium fluorescein was corrected for the dilution occurring because of sample removal and replacement of the volume with fresh buffer: concentration c = (c_t−1_ × V_s_ + c_t_ × V_K_)/V_K_, with c_t_ being the measured concentration, c_t−1_ the measured concentration of the previous period, V_s_ = sample volume, V_K_ = volume of Ussing chamber. Flux was calculated as J = (c_t_−c_t−1_) × V_K_/Δt × A with Δt representing the duration of the measurement period and A being the tissue area. Finally, the apparent permeability P_app_ = J/c_a_ was computed, with c_a_ being the measured fluorescein concentration in the apical side of the Ussing chamber.

### Real-time quantitative polymerase chain reaction

Subsequent to Ussing chamber experiments, samples for quantitative real-time polymerase chain reaction (qPCR) were taken from the chambers. The tissue specimens were rinsed, transferred into RNA*later* RNA Stabilization Reagent (Qiagen GmbH, Hilden, Germany), and stored at −20°C until RNA isolation, the determination of RNA quantity and quality, and cDNA synthesis. These steps were performed as described recently (Lodemann et al., 2017). The iScript cDNA Synthesis Kit was employed to reverse-transcribe 100 ng total RNA of each sample to cDNA (Bio-Rad Laboratories GmbH, Munich, Germany) according to the manufacturer's recommendations, and primers were obtained from Eurofins MWG Synthesis GmbH (Ebersberg, Germany). Primer sequences were claudin-4 (CLDN4, *Sus scrofa*) (Sense) 5′-CAA CTG CGT GGA TGA TGA GA-3′, (Antisense) 5′-CCA GGG GAT TGT AGA AGT CG-3′; and beta-2-microglobulin (B2M, *Sus scrofa*) (Sense) 5′-AAACGGAAAGCCAAATTACC-3′, (Antisense) 5′-ATC CAC AGC GTT AGG AGT GA-3′. Real-time quantitative PCR was performed in the iCycler iQ Real-Time PCR Detection System (Bio-Rad Laboratories GmbH, Munich, Germany) by using SYBR green I detection. The reactions were performed as triplicates; the final volume (15 μl) contained iQ SYBR Green Super mix (Bio-Rad Laboratories GmbH, Munich, Germany), primers (0.3 μl of 20 pmol/μl each), and 5 μl cDNA. iQ5 software (Bio-Rad Laboratories GmbH, Munich, Germany) was employed for the calculation of the relative amount of target genes in PP FAE tissue specimens compared with villous epithelia. For normalization, the geometric mean of the reference gene (B2M) was used.

### Protein extraction, immunoblot, and densitometry

Tissue samples were frozen in liquid nitrogen. Homogenization was carried out in a Tris-buffer containing in mmol·l^−1^: Tris (10), NaCl (150), Triton X-100 (0.5), SDS (0.1), and a set of protease inhibitors (Complete, Boehringer, Mannheim, Germany). The supernatant was cooled on ice for 30 min after centrifugation for 1 min at 13,000 rpm (Eppendorf centrifuge 5418, Eppendorf AG, Hamburg, Germany). Samples were then centrifuged for 15 min at 15,000 x g at 4°C (sigma 3-30ks, Sigma-Aldrich, Munich, Germany). Quantification followed by using a BCA protein assay reagent (Pierce, Rockfort, Il, USA) and a plate reader (EnSpire Multimode Plate Reader, Perkin Elmer, USA). Protein (20 μg), and Laemmli buffer (Bio-Rad Laboratories GmbH, Munich, Germany) were mixed and loaded onto a 12.5% polyacrylamide gel for electrophoresis. Samples were then transferred onto a PVDF membrane. Subsequently, membranes were blocked for 60 min in 5% milk (in Tris-buffered saline with 0.1% Tween 20) and incubated with antibodies raised against claudin-1, -2, -3, -4, -5, and -8 and β-actin (Life Technologies, USA) by employing 1 μg/ml in accordance with the manufacturer's instructions, respectively. Later peroxidase-conjugated goat anti-mouse and anti-rabbit antibodies (Cell Signaling Technology, Danvers, MA, USA) were used to bind the primary antibodies. Signals were detected via the chemiluminescence detection system of Clarity Western ECL Blotting Substrate (Bio-Rad Laboratories GmbH, Munich, Germany) and a luminescence imager (ChemiDoc MP, Munich, Germany). Densitometric analysis of the specific bands was performed by using the imager associated software Image Lab. Finally, the measured values were expressed as a ratio to the relevant β-actin band of the same sample. Calculated values for the VE were set to 100%, and the matching PP-values were expressed in relation. The calculated correction factor derived from morphometric analysis was used to account for the different number of epithelial cell contacts.

### Immunohistochemistry

Tissue samples were fixed in 2% paraformaldehyde diluted in PBS (16% paraformaldehyde, E15700, Science Service, Munich, Germany) for 2 h and kept in PBS until dehydration, embedded in paraffin, and cut into sections (Leica RM 2245 microtome, Leica Microsystems Heidelberg, Germany). For staining, the paraffin was removed by using a decreasing xylol-ethanol gradient. Heat-induced epitope retrieval was carried out for 20 min in EDTA or citrate buffer. Sections were then blocked for 30 min in 5% goat serum in PBS and incubated with antibodies raised against claudin-1, -2, -3, -4, -5, and -8 (1:100, Life Technologies, USA) for 60 min at 37°C or overnight at 4°C. After being washed with PBS, samples were incubated with goat anti-rabbit Alexa Fluor-488, goat anti-rabbit Alexa Fluor-594, or goat anti-mouse Alexa Fluor-488 (1:1,000, Life Technologies, USA). Nuclei were stained by using DAPI (1:5,000) before the specimens were mounted with ProTags Mount Flour (Biocyc, Luckenwalde, Germany). In accordance with the manufacturer's instructions, control staining was performed by employing matching IgG and IgG1 isotype controls and with secondary antibodies only. Hematoxylin and eosin staining was performed as previously described (Amasheh et al., 2009). Slides were analyzed by using Leica microscopes of the DMI 6000 and TCS SP2 series (Leica Microsystems Heidelberg, Germany).

### Statistical analysis

The data are expressed in means and standard error of the mean (SEM), where n represents the number of animals used. Statistical analysis was carried out by using Student's *t*-test. Values below *p* = 0.05 were considered to be statistically significant, being denoted as ^*^p < 0.05, ^**^p < 0.01, and ^***^p < 0.001.

## Results

### PP FAE tissue specimens show stronger barrier properties

**Figure 1:**
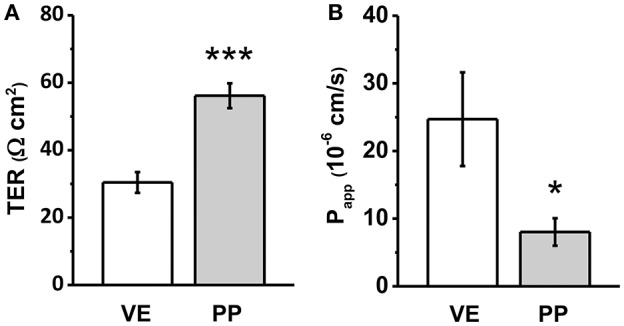
**(A)** Transepithelial resistance (TER), and **(B)** Apparent permeability (P_app_) for the paracellular flux marker fluorescein of Peyer's patch follicle associated epithelium (PP) compared to neighboring villous epithelium (VE). Whereas higher TER values were detected in PP FAE, lower paracellular permeability for fluorescein was observed consistently (*n* = 7, ^*^p < 0.05, ^***^p < 0.001).

Electrophysiological measurements revealed significantly higher TER in PP tissue, when compared with adjacent VE. VE TER values of 30.4 ± 3.1 Ω cm^2^ represented the rather leaky type of jejunal epithelium, whereas in adjacent PP, a two-fold higher TER was observed (56.1 ± 3.7 Ω cm^2^, ^***^p < 0.001, *n* = 7; Figure 1A).

Experiments employing sodium fluorescein (332 Da) as the paracellular marker revealed an even more pronounced difference of apparent permeability, being calculated from flux measurements as a three-fold lower value (Figure 1B: VE: 24.7 ± 6.92 10^−6^ cm/s, PP FAE: 8.03 ± 2.03 10^−6^ cm/s, ^*^p < 0.05, *n* = 7).

### Morphometric analysis reveals a PP surface correction factor of 2.3

**Figure 2:**
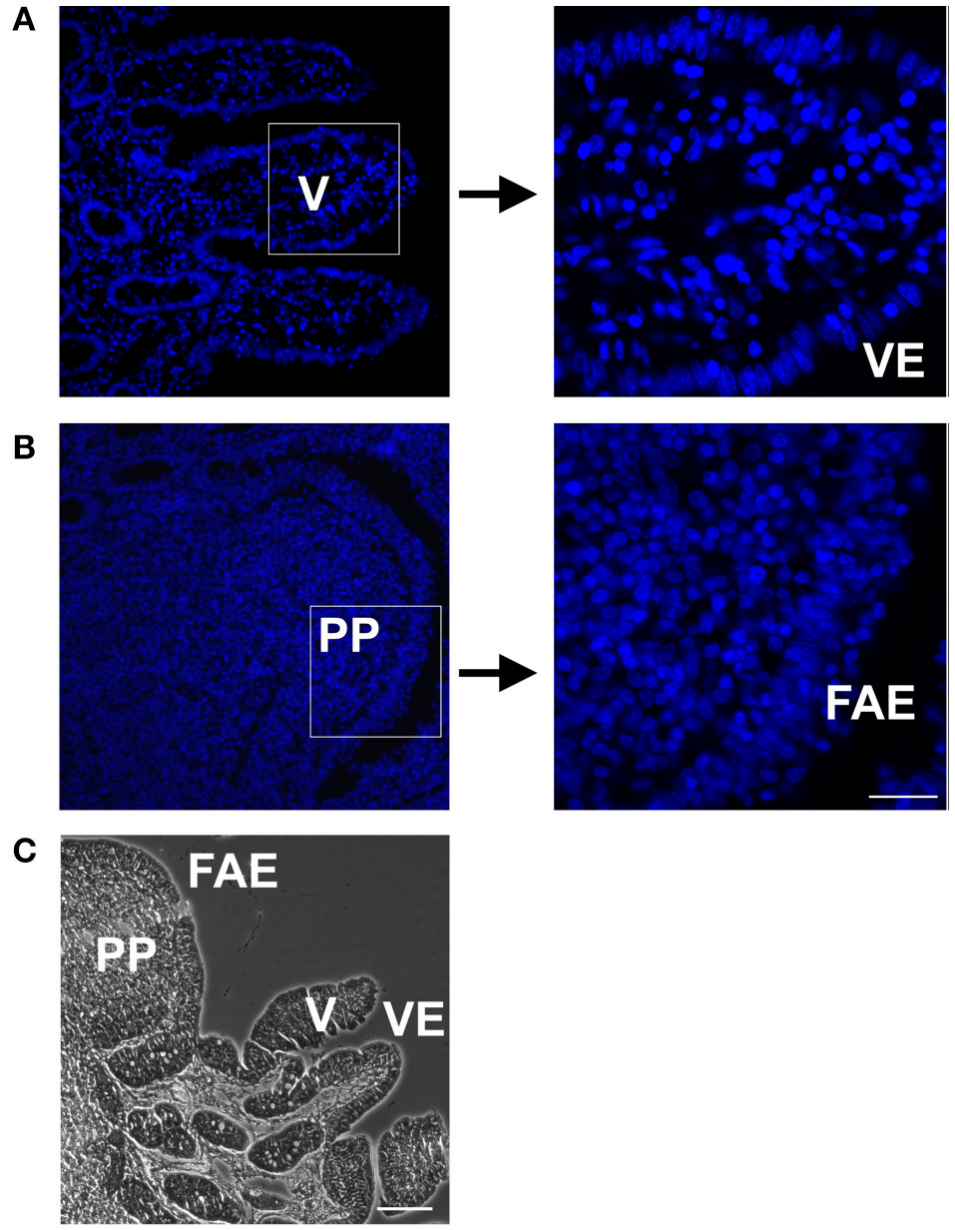
DAPI staining of Peyer's patch follicle associated epithelium (PP FAE) compared to neighboring villi (V) with villous epithelium (VE), and Hematoxylin and Eosin (HE) staining. **(A)** VE **(B)** PP FAE Tissue specimen; magnification 20×, marked squares are shown in 63× magnification **(C)** HE staining representing PP with FAE, and neighboring V with VE (bar: 50 μm).

Histological analyses revealed major differences of PP compared with neighboring VE (Figure 2). Because of the different morphology between the PP and VE, a surface correction for the proper comparison of quantitative epithelial protein became necessary. Whereas, the serosal structure and area were equal in both the PP FAE and VE, the PP had a lower cell count and therefore fewer fraction of TJ proteins. This had to be taken into account when analyzing the quantitative results of immunoblotting data. Since the FAE covering PP is interrupted by interfollicular villi, the ratio between the two areas also had to be included when calculating the correction factor.

Surface correction was performed in three separate steps, namely (i) correction for interfollicular areas, (ii) correction by cell count, and (iii) calculation of ratio. With regard to step (i), the morphology of PP in piglets aged 2 months has been previously researched intensively (Barmann et al., 1997). The average width and length of PP has been measured at 0.71 mm and 0.86 mm (Barmann et al., 1997), giving it a total area of 0.6106 mm^2^. Assuming a round shape, we calculated the average diameter of PP to be 0.88 mm. Since the average distance (0.11 mm) between PP was also provided in the data of Barmann et al. (1997), we calculated the average diameter of PP and the surrounding VE as being 0.99 mm, giving it an area of 0.77 mm^2^. Because of our Ussing chamber setup, we used an area of 95 mm^2^ for measurements; this can also be expressed as roughly 123 PP plus surrounding VE. Combining the average size of PP and the given number, a total area of 75.10 mm^2^ of the tested area of 95 mm^2^ can be estimated to be covered by PP, whereas the remaining 19.90 mm^2^ is interfollicular epithelium. Expressed in percentages, we calculated the ratio of FAE to VE in PP to be 79% : 21%. For point (ii), the DAPI staining of PP and VE was analyzed via immunofluorescence microscopy (Leica DMI 6000 B, Leica, Germany). Cell count was performed at 63× magnification. Measurements of PPs revealed that FAE had 34 ± 2 cells per 100 nm length, whereas the VE revealed 120 ± 8 cells per 100 nm. Because of the presence of interfollicular villi in PP, the total amount of cells had to be reduced by the percentage calculated in step (i). Therefore, 21% of the area was covered by 120 cells/100 nm, whereas 79% was covered by 34 cells/100 nm, resulting in a combined cell count of 52 cells/100 nm for PP tissue. In step (iii), these cell counts gave a ratio of 2.3, which was employed as a correction factor for the proper comparison of immunoblotting data, as outlined below.

### Immunoblot detection of claudins

**Figure 3:**
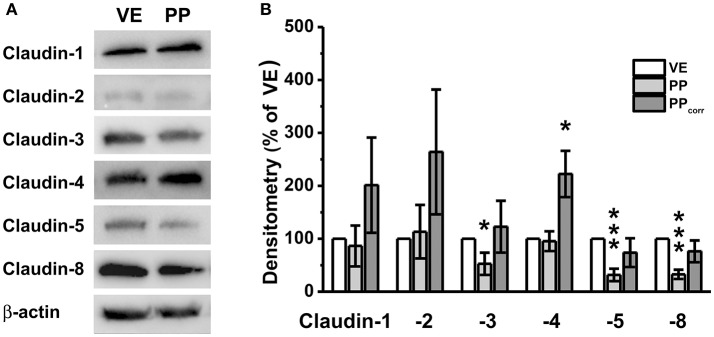
**(A)** Immunoblots, **(B)** Densitometry. **(A)** In both, Peyer's patch (PP) and neighboring villous epithelium (VE), claudin-1, claudin-2, claudin-3, claudin-4, claudin-5, and claudin-8, were detected. **(B)** Densitometric analysis of tight junction protein signals revealed significantly stronger claudin-4 expression in PP after surface correction (*n* = 5, ^*^p < 0.05, ^***^p < 0.001).

Immunoblotting revealed specific signals of claudin-1, -2, -3, -4, -5, and -8 in all preparations (Figure 3A). Without surface correction, densitometric analyses comparing specific signals in the VE and PP tissue showed weaker signals of single members of the claudin family including claudin-3, -5, and -8, whereas claudin-1, -2, and -4 were not significantly different from VE. The values were corrected by taking the different morphology and the number of cellular connections into account, by employing the calculated correction factor of 2.3, as described above. Whereas corrected values revealed no significant differences of PP expression compared with neighboring tissue for claudin-1, -2, -3, -5, and -8, a markedly stronger expression was observed for the tightening or sealing TJ protein claudin-4. Although the VE-value was normalized to 100 ± 0%, densitometric evaluation of PP samples revealed comparable expression levels (95.4 ± 18.7%). Surface correction employing the calculated factor (×2.3) led to a final value of 222.3 ± 49% (^*^p < 0.05, *n* = 5; Figure 3B).

### Immunohistochemistry

**Figure 4:**
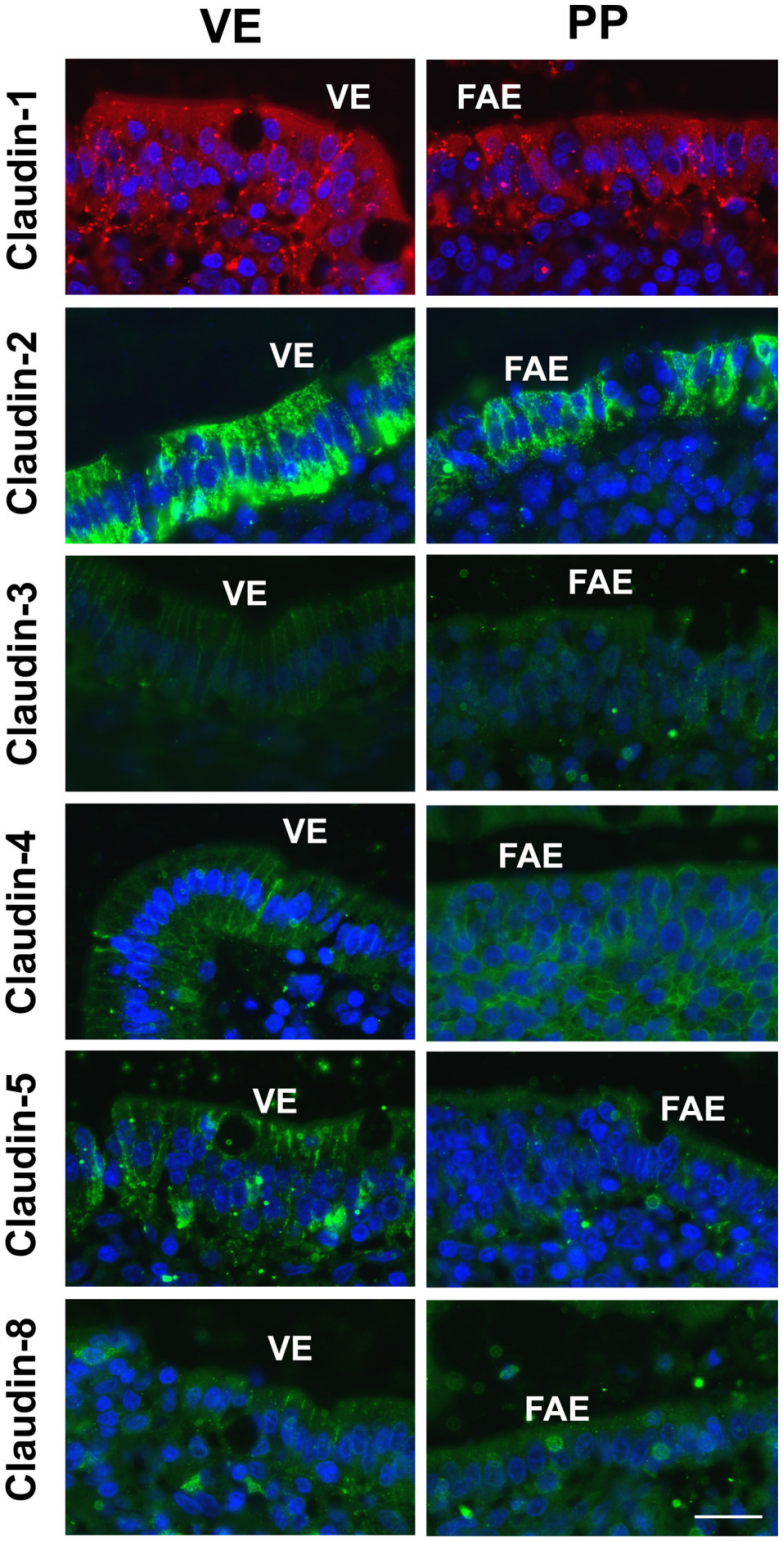
Immunofluorescent staining revealed signals of all claudins within epithelial cells in Peyer's patch follicle-associated epithelium (PP FAE) and neighboring villous epithelium (VE). The majority of claudins was detected in the apicolateral membrane, including claudin-4 in PP FAE (*n* = 5, respectively, bar: 20 μm).

Immunohistological analyses revealed focused expression and localization of claudins within the TJ complexes of both the PP FAE and adjacent VE in single staining (Figure 4). The variable signal intensities in the heterogeneous cell populations of porcine tissue specimens did not allow proper quantification or colocalization analysis. However, in order to confirm specificity, control staining was performed by employing matching IgG and IgG1 isotype controls or secondary antibodies only; no specific TJ signals were detected in epithelial cells, respectively (not shown).

### Real-time quantitative polymerase chain reaction

**Figure 5:**
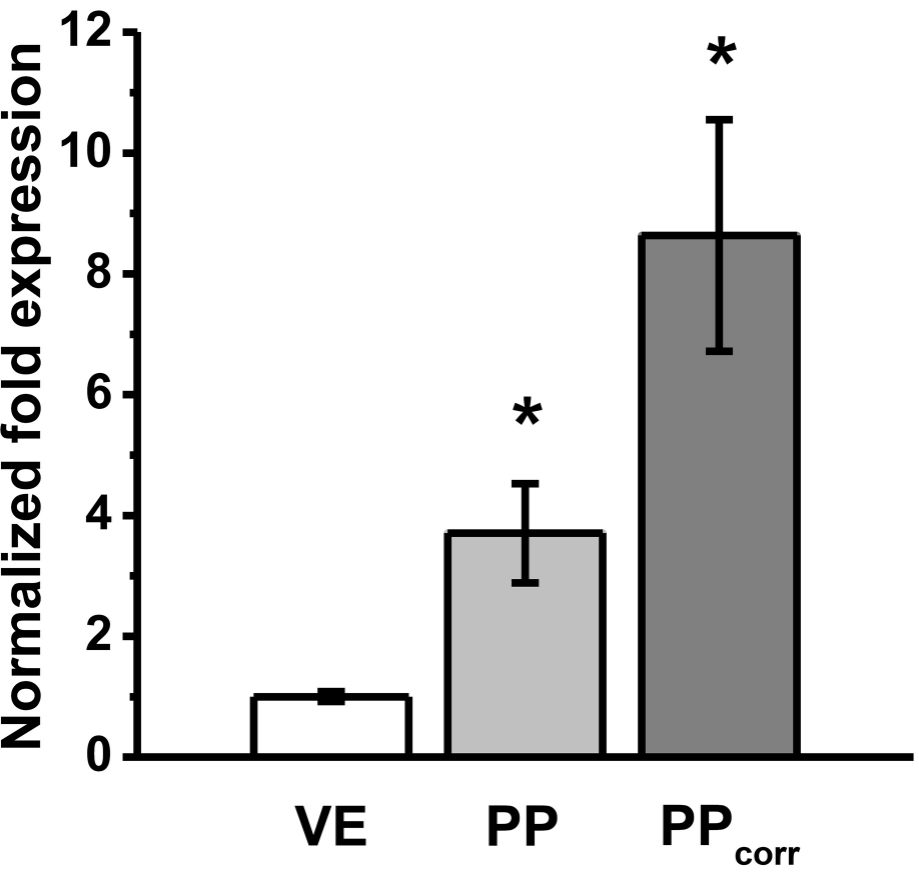
RT-qPCR revealed increased levels of mRNA expression of claudin-4 in Peyer's patch follicle associated epithelium (PP FAE) tissue specimens vs. villous epithelium (VE). Normalized fold expression was calculated by the ΔΔCt method. ^*^P < 0.05, *n* = 3, respectively.

To analyze the expression of claudin-4 further, RT-qPCR was performed, revealing increased levels of mRNA in PP FAE tissue specimens vs. VE and after surface correction (PP_corr_). Normalized fold expression was calculated by the ΔΔCt method (^*^p < 0.01, *n* = 3; Figure 5).

## Discussion

Organized as bands surrounding the upper part of epithelial cells, TJs are the structural correlate of intestinal barrier function (Martinez-Palomo and Erlij, 1975). In recent years, our understanding of the molecular basis of epithelial barrier function has greatly advanced (Markov et al., 2015, 2017; Suzuki et al., 2017). TJ strands are composed mainly of tetraspan transmembrane proteins of the claudin family (Furuse et al., 1998) and determine the paracellular movement of ions, water, and small molecules (Amasheh et al., 2002; Rosenthal et al., 2010). The extracellular loops of various claudins interact to form a paracellular seal or gap that determines paracellular permeability (Colegio et al., 2002). Our structural understanding of this principle has been enlightened by the recently reported crystallographic structure of a claudin (Suzuki et al., 2014).

This study aimed to characterize the barrier of porcine PP FAE. Since the postnatal development of PP is strongly influenced by contact with living microbial antigens (Barmann et al., 1997), a tight paracellular seal is essential for the correct presentation of antigens through M cells to the underlying follicle immune cells and therefore for the proper maturation of the immune system.

In adult pigs, various PP are found in the distal jejunum with an average length of 3.8 cm. Moreover, a continuous ribbon-like patch can be found in the ileum (Kararli, 1995). Although age, body weight, and length of the small intestine have been shown to have a positive influence on the number of PP (Kararli, 1995), information about the paracellular barrier of the FAE is limited. Nevertheless, it can be expected to play a major functional role, because of its exceptional differentiation with regard to barrier properties and specific claudin expression, potentially also affecting mechanisms of stress-induced barrier disruption. These mechanisms involve corticotrophin releasing hormone, acetylcholine, substance P, and mast cells and even the selectivity of immune responses compared with the defined receptor-mediated transcellular processes currently discussed for M cells within FAE with allograft inflammatory factor 1 promoting transcytosis, which might be compromised by the paracellular pathway (Keita et al., 2010; Kishikawa et al., 2017). Therefore, the results of our study will apply to a broad research field in porcine and human pathophysiology.

In accordance with our previous study focusing on rat PP (Markov et al., 2016), the current study has revealed claudins in all tissue specimens of the porcine PP FAE and neighboring villous epithelia. However, among the major intestinal TJ proteins, only claudin-4 shows a significantly higher expression in porcine PP FAE. The significance of the similar findings in the pig system compared with our recent study in rat regarding claudin-4 and barrier properties (Markov et al., 2016) strongly suggests that a major common denominator has now been identified on both, protein and mRNA level. The pig system closely resembles the human intestine and therefore is a useful model for studying the detailed principles of PP FAE function and regulation in barriology and immunology, which are currently being discussed in the context of formula feeding and food allergies (Chen et al., 2014; Wu et al., 2015; Yeruva et al., 2016). However, as a refinement, current limitations regarding the visualization of co-localization and the quantification of immunohistological images might be overcome in epithelial monolayer and co-culture models.

Within intestinal TJ, claudin-4 plays an eminent role, as it can be affected by a wide variety of factors, including secondary plant compounds, such as the flavonoid quercetin (Amasheh et al., 2008), bacteria and bacterial toxins, e.g., enter toxigenic *Escherichia coli* and cholera toxin (Markov et al., 2014; Lodemann et al., 2017). Thus, susceptibility to barrier strengtheners and pertubators might be attributed to claudin-4, as demonstrated in detail for *Clostridium perfringens* enterotoxin (Sonoda et al., 1999; Shinoda et al., 2016). The first evidence of a direct influence of a claudin in the selective control of paracellular ion permeability was obtained by Van Itallie et al. (2001) for claudin-4. The direct association of decreased claudin-4 expression has been demonstrated for a variety of intestinal pathophysiological conditions, including the inflammatory bowel diseases collagenous colitis, ulcerative colitis, and Crohn's disease (Bürgel et al., 2002; Prasad et al., 2005; Das et al., 2012). Thus, a higher expression might be generally regarded as a preventive or protective mechanism.

Apart from claudin-4, a number of other intestinal claudins are known to be susceptible to barrier effectors and perturbation, namely claudin-1 and claudin-2 (Amasheh et al., 2009, 2010), claudin-3 and claudin-4 (Markov et al., 2014), claudin-5 and claudin-8 (Dittmann et al., 2014; Barmeyer et al., 2017). Although the interplay of these TJ proteins with transport function and signaling might still be worth considering with regard to porcine PP FAE (Markov et al., 2015, 2017), no differences of these claudins with respect to their localization and expression levels have been observed, at least under the non-challenging conditions of the current study.

In porcine PP FAE, claudin-4 can be regarded to strengthen the sealing of the paracellular pathway and, therefore, to improve the selectivity of the transcellular pathway. This mechanism would allow a stronger focus on transcellularly presented antigens and less susceptibility for barrier perturbation as a preventive mechanism. If barrier effectors selectively impair porcine PP FAE function during pathophysiological events, they might also affect intestinal immune defense, but this remains to be elucidated. The outcome of our study, however, lays the foundation for focused approaches with regard to specific effects on PP FAE in intestinal physiology and pathophysiology.

## Ethics statement

This study was specifically reviewed and approved by Berlin State Ethics Committee (Ethik-Kommission des Landes Berlin) and therefore was carried out in accordance with the German law for the care and use of experimental animals. Administrative responsibility rests with the Berlin State Department for Health and Social Affairs (Landesamt für Gesundheit und Soziales Berlin); Approval No. G 0321/13.

## Author contributions

All authors have read and approved the manuscript. JR and SA designed, planned, and supervised the experiments and wrote the article. JR, EF, AM, and SA performed the experiments and data analysis.

### Conflict of interest statement

The authors declare that the research was conducted in the absence of any commercial or financial relationships that could be construed as a potential conflict of interest.
